# Mason-Pfizer Monkey Virus Envelope Glycoprotein Cycling and Its Vesicular Co-Transport with Immature Particles

**DOI:** 10.3390/v10100575

**Published:** 2018-10-20

**Authors:** Petra Grznárová Prokšová, Jan Lipov, Jaroslav Zelenka, Eric Hunter, Hana Langerová, Michaela Rumlová, Tomáš Ruml

**Affiliations:** 1 Department of Biochemistry and Microbiology, University of Chemistry and Technology, 166 28 Prague, Czech Republic; petra.grznarova@natur.cuni.cz (P.G.P.); jan.lipov@vscht.cz (J.L.); jaroslav.zelenka@vscht.cz (J.Z.); 2 Imaging methods core facility at BIOCEV, Faculty of Science, Charles University, 252 50 Prague, Czech Republic; 3 Emory Vaccine Center at the Yerkes National Primate Research Center, Emory University, Atlanta, GA 30329, USA; ehunte4@emory.edu; 4 Department of Biotechnology, University of Chemistry and Technology, 166 28 Prague, Czech Republic; langeroh@vscht.cz (H.L.); michaela.rumlova@vscht.cz (M.R.)

**Keywords:** Mason-Pfizer monkey virus, envelope, intracellular trafficking, endosomes, virus-like particles, transport

## Abstract

The envelope glycoprotein (Env) plays a crucial role in the retroviral life cycle by mediating primary interactions with the host cell. As described previously and expanded on in this paper, Env mediates the trafficking of immature Mason-Pfizer monkey virus (M-PMV) particles to the plasma membrane (PM). Using a panel of labeled RabGTPases as endosomal markers, we identified Env mostly in Rab7a- and Rab9a-positive endosomes. Based on an analysis of the transport of recombinant fluorescently labeled M-PMV Gag and Env proteins, we propose a putative mechanism of the intracellular trafficking of M-PMV Env and immature particles. According to this model, a portion of Env is targeted from the trans-Golgi network (TGN) to Rab7a-positive endosomes. It is then transported to Rab9a-positive endosomes and back to the TGN. It is at the Rab9a vesicles where the immature particles may anchor to the membranes of the Env-containing vesicles, preventing Env recycling to the TGN. These Gag-associated vesicles are then transported to the plasma membrane.

## 1. Introduction

Mason-Pfizer monkey virus (M-PMV) is a D-type retrovirus that preassembles its immature particles in the cytoplasm of infected cells [1,2]. Due to both temporally and spatially separate steps of assembly and budding, M-PMV has been utilized as a model to study the retroviral life cycle with potential extrapolation to more complex viruses, e.g., human immunodeficiency virus (HIV). 

As in other retroviruses, immature M-PMV particles assemble from the polyprotein precursors of structural proteins and enzymes: Gag, GagPro, GagProPol in defined ratios [3]. All three polyproteins initiate synthesis on free polysomes in the cytoplasm, but are co-translationally transported to the microtubule organizing center (MTOC) by the cellular molecular motor dynein. This requires the interaction of a cytoplasmic targeting/retention signal (CTRS) in the matrix domain of Gag and a light chain of dynein (Tctex-1) [4,5,6]. The MTOC region seems to be an ideal place for this intracellular assembly as it anchors many organelles and factors, including chaperonins, which may facilitate this process. It was shown that interaction with the chaperonin TRiC stabilizes Gag and facilitates its multimerization [7]. The multimerization seems to be initiated by conformational changes of Gag induced by its specific interactions with unspliced viral mRNA [8]. The precursor of the envelope glycoprotein (Env) is translated from spliced mRNA on the rough endoplasmic reticulum (ER) and it is co-translationally transported to the lumen of the ER where it trimerizes and is partially glycosylated. The glycosylation is completed in the Golgi apparatus, which is also the site where the cellular protease furin cleaves Env into two subunits, gp70 (surface subunit) and gp22 (transmembrane subunit), which remain associated. A detailed explanation of the molecular mechanism of intracellular transport of M-PMV Env has been hindered by the lack of specific anti-Env antibody. Therefore, the detection of physiological levels of Env in M-PMV infected cells is limited to the use of polyclonal antibodies recognizing all M-PMV proteins.

In general, Env, as an integral type-I membrane protein, would be expected to be targeted from the TGN via the secretory pathway to the plasma membrane (PM). The incorporation of Env into budding particles does not appear to take place at the PM, but requires recycling since the mutation that blocks endocytosis also inhibits Env incorporation [9]. After Env is endocytosed from the PM, it is trafficked to recycling endosomes, which were suggested to be the location of initial Env/Gag interactions [10]. This interaction would then mediate the trafficking of Env and immature capsids-associated vesicles back to the PM where budding is initiated.

Here we present a follow-up study focused on the Env/Gag trafficking pathway. The intracellular transport of Env was monitored using a construct encoding the whole M-PMV genome where the transmembrane domain (TM) of Env is tagged with the mCherry fluorescent protein (mCherryTM). Live-cell imaging experiments showed that a majority of mCherry is localized to intracellular vesicles of unknown origin. We used a panel of endosomal markers based on RabGTPases tagged with enhanced green fluorescent protein (EGFP) to perform colocalization studies with mCherryTM. This approach proved that mCherryTM is localized to Rab7a endosomes and to a lesser extent to those positive for Rab9a. Live-cell imaging data showed the joint transport of mCherryTM-containing vesicles and EGFP-labeled Gag. Both signals were detected in a pericentriolar region as well as at the cell periphery. The results allowed us to propose a mode of intracellular trafficking of M-PMV Env and its site(s) of interaction with Gag immature particles.

## 2. Materials and Methods 

### 2.1. Plasmids

The plasmids pSARMXmCherryTM (encoding whole M-PMV proviral DNA with mCherry coding sequence inserted into the extracellular region of the transmembrane domain, TM, immediately adjacent to the membrane-spanning domain [11]) and pEGFP-Rab5a were kindly provided by Dr. Paul Spearman (Cincinnati, OH, USA). pSARM-Gag-EGFP-M100A was constructed previously and is described in Clark et al. [12]. The plasmids pEGFP-Rab7a, pEGFP-Rab9a and pEGFP-Rab11a were kindly provided by Richard Pagano (Addgene plasmids # 12605, # 12663 and # 12674) [13].

### 2.2. The Introduction of I18A and Y22A Mutations into pSARMXmCherryTM Construct

It was shown previously that Env proteins mutated in the cytoplasmic domain (cytoplasmic tail, CT) of TM at positions 18 and 22 (I18A and Y22A) were efficiently transported to the plasma membrane, but failed to be incorporated into the released virions; suggesting that critical step(s) in the trafficking of these proteins are perturbed [9,14]. The following primers were designed to introduce I18A into pSARMXmCherryTM. Two sets of primers were designed to amplify two halves of the plasmid carrying the mutation: 

Set 1:

5′-AAACCTGCACAAGTCCATTATCATCGCCTTG and
5′-AAGAGGGCCCAATATCCGAGCAAAGACG

Set 2

5′-GACTTGTGCAGGTTTGGCCTGGATGCTC and
5′-ATATTGGGCCCTCTTATCAGCAAGGCCTGG

To regenerate the plasmid, the two polymerase chain reaction (PCR) products, which had complementary 15 nucleotides ends, were joined using InFusion^®^ according to the manufacturer’s instructions. After incubation at 50 °C for 15 min, the mixture was transformed into *E. coli* JM109 competent cells and incubated at 30 °C.

An identical approach was used for the Y22A mutant form of pSARMXmCherryTM.

Set 1:

5′-GTCCATGCTCATCGCCTTGAACAAGAAGACAGTGG and
5′-AAGAGGGCCCAATATCCGAGCAAAGACG

Set 2

5′-GCGATGAGCATGGACTTGTATAGGTTTGGC and
5′-ATATTGGGCCCTCTTATCAGCAAGGC

### 2.3. Cultivation of Cells, Transfection of Plasmids and Imaging

All transfections were performed in COS-1 cells [CRL-1650]—African green monkey kidney fibroblasts transformed with SV40 (ATCC, Manassas, VA, USA). The cells were cultivated in Dulbecco’s modified Eagle’s medium (DMEM) supplemented with 10% fetal serum. For all imaging studies, the cells were seeded and grown on sterile coverslips placed in 6-well plates. The transfection was performed usually 24 h later using FugeneHD (Promega, Fitchburg, WI, USA) reagent in a ratio of 3 μL of reagent to 1 µg of DNA. The transfection mixture was prepared according to the manufacturer’s instructions in 100 µL of OptiMem^®^ (Thermo Fisher Scientific, Waltham, MA, USA) media per well and added dropwise to the cells.

For the RabGTPase colocalization studies, 100 ng of RabGTPase DNA was mixed with 1 µg of pSARMXmCherryTM plasmid variant and transfected as described above. Twenty-four hours post-transfection, the cultivation medium was replaced with fixative (4% formaldehyde in phosphate buffered saline (PBS)) and incubated for 20 min at room temperature, washed with PBS, incubated with 50 mM NH_4_Cl for 5 min (to remove residual traces of formaldehyde) and again washed with PBS. The coverslips were mounted in Vectashield mounting medium (Vector Laboratories, Burlingame, CA, USA).

For studies of the membrane localization of native Env (of untagged virus), 1 µg of pSARMX construct or pTML plasmid was mixed with 3 µL of FugeneHD and transfected as above. At 24 h post-transfection, the coverslips with the living cells were placed on ice and incubated with goat anti-M-PMV antibody for 25 min to stain surface Env. To visualize the intracellular content, fixation was performed as described above. After the fixation, 1% bovine serum albumin (BSA) in PBS was added to the samples to block nonspecific interaction sites. After 20 min of incubation the cells were permeabilized using 0.1% Tween 20 in PBS for 10 min and then incubated with rabbit anti-CA antibody [15] for 30 min at room temperature. To visualize Env-bound primary antibody, the cells were incubated with secondary anti-goat IgG antibody conjugated with AlexaFluor^®^ 350 (Invitrogen, Carlsbad, CA, USA) for 20 min at RT. To visualize immature capsids, the cells were incubated with secondary anti-rabbit IgG antibody conjugated with fluorescein isothiocyanate (FITC) under the same conditions. After multiple washes, the coverslips were mounted into Vectashield mounting medium.

For studies of the membrane localization of mCherry tagged Env, 1 µg of pSARMXmCherryTM wild-type (WT) or mutant variant construct was mixed with 3 µL of FugeneHD and transfected as above. After 48 h, the living cells were incubated with goat anti-M-PMV antibody or rabbit anti-mCherry antibody (ab167453, Abcam, Cambridge, UK) on ice for 25 min. After the formaldehyde fixation, 1% BSA in PBS was added to the samples to block nonspecific interaction sites and samples were immunostained with secondary anti-goat IgG antibody conjugated with Alexa Fluor^®^ 350 (Invitrogen, USA) or with secondary anti-rabbit IgG antibody conjugated with Alexa Fluor^TM^ Plus 488 (Thermo Fisher Scientific, Waltham, MA, USA) for 20 min at RT. After multiple washes, the coverslips were mounted into Vectashield mounting medium (with DAPI for anti-mCherry antibody staining). 

For the study of the colocalization of cis/medial Golgi and mCherryTM, COS-1 cells were transfected with 1 µg of pSARMXmCherryTM WT or mutant construct as it is described above. 48 h later were cells fixed with 4% formaldehyde, blocked with BSA and permeabilized as described above. All samples were incubated with primary antibody rabbit anti-GOLM1 (Sigma Aldrich, St. Louis, MO, USA) and after multiple washes immunostained with secondary anti-rabbit IgG antibody conjugated with Alexa Fluor^TM^ Plus 488 and mounted into Vectashield medium. 

All fixed samples were imaged using an Olympus cell^R microscope (Olympus, Tokyo, Japan) or spinning disk confocal microscope (Andor, Belfast, UK).

### 2.4. Immunolabeling of Viral Proteins with Radioactive Isotopes and Immunoprecipitation (Pulse Chase Assay)

For this assay, the cells were seeded into 6-well plates in multiple sets. They were transfected using 1 µg of DNA (pSARMX-WT, pSARMXmCherryTM-WT or mutant variants) with 3 µL of FugeneHD in 100 µL of OptiMem^®^ medium per well and incubated for 48 h. The cultivation medium was then replaced with DMEM medium lacking methionine and cysteine. After 15 min of incubation, the cells were pulse-labeled with 100 µCi of [^35^S]-labeling mix (Isolabel [^35^S]; a mixture of [^35^S] methionine and [^35^S] cysteine in the ratio 4:1) per well for 30 min at 37 °C under physiological conditions. One set of samples was lysed immediately (pulse/cell lysates) and others after 2 h, 4 h and 8 h of incubation in complete DMEM. The culture media were centrifuged at 13,000 RCF for 5 min at room temperature. Lysis buffer A (8.76 g/L NaCl, 6.06 g/L Trizma base, 10 mL/L Triton X-100, 10 g/L sodium deoxycholate) was added and samples were incubated for 10 min, placed on ice for 5 min, centrifuged at 13,000× *g* RCF for 2 min and supernatants were transferred into new tubes. The concentration of sodium dodecyl sulfate (SDS) was adjusted to 0.1% in both culture media and cell lysates and all the samples were incubated with a suspension of inactivated *Staphylococcus aureus* (*Staph A*) at room temperature for 30 min to remove unspecific binders. 1 µL of goat anti-M-PMV antibody was added to each sample after the pellet of *Staph A* was removed and incubated overnight at 4 °C. The immunocomplexes were precipitated with *Staph A* in lysis buffer B (Lysis buffer A + 1 g/L SDS). Viral proteins were resolved by Tris-Tricine SDS polyacrylamide gel electrophoresis (SDS PAGE). Gels were incubated in drying solution (100 mL/L acidic acid, 400 mL/L methanol, 30 mL/L) for 20 min and dried in a gel dryer (Bio-Rad, Hercules, CA, USA) for 2 h at 80 °C. The imaging screen (Molecular Imager FX™ Imaging screen-K) was exposed to the dried gels for one week.

### 2.5. Live-Cell Imaging

For live-cell imaging, the cells were seeded into glass-bottomed dishes (MatTek Corporation, Ashland, MA, USA) and incubated in complete DMEM. 

To determine the localization of mCherryTM protein variants, COS-1 cells were transfected with 1 µg of pSARMXmCherryTM WT or mutated variant with 3 μL of FugeneHD in 100 µL of OptiMem^®^ medium per dish. 

For studies of the co-transport of mCherryTM and EGFP-labeled immature capsids, the cells were co-transfected with a mixture of 200 ng of pSARM-Gag-EGFP-M100A and 800 ng of pSARMXmCherryTM WT (or mutant variant) and 3 µL of FugeneHD in 100 µL of OptiMem^®^ media per dish. We showed previously that the pSARM-Gag-EGFP-M100A vector produced minimal levels of Env, likely due to a decrease in the splicing efficiency of Env mRNA since some of the potential splice branch points for Env splicing were removed during the codon-optimization of the Gag sequence.

Immediately before imaging, the culture media in all samples were replaced with phenol red lacking DMEM supplemented with 10% FBS, L-glutamine (final concentration 0.584 g/L) and 1% vitamins solution. The samples were then placed into the incubation cell of an Olympus cell^R microscope and incubated at physiological conditions while the videos were acquired. All videos were captured each second with exposure of 100 ms.

### 2.6. Quantification of Colocalization 

Colocalization of Rab-positive vesicles and mCherryTM was analyzed with ImageJ (version 1.52g, National Institutes of Health, Bethesda, MD, USA) [16,17,18]. Due to the unusual type of colocalization, where both signals do not overlap, since the Rab signal is on the surface of the endosome and the mCherry signal appears to fill the inner volume of the endosome, a mask of regions of interests (ROIs) was created based on the Rab signal. This mask was applied to the mCherry channel image and the intensity of the mCherry signal was measured in all ROIs. This approach gave a yes/no answer as to whether particular ROI (or actually Rab-positive endosome) also contained an mCherry signal or only the background signal. Based on these results, the percentage of mCherry-positive Rab-positive endosomes from the total number of Rab-positive endosomes was identified.

The quantification of the colocalization of mCherryTM variants and GagEGFPWT was performed by Coloc2 analysis in ImageJ (version 1.52g, National Institutes of Health, Bethesda, MD, USA) [19,20,21]. The analysis was done on the first image from the time-lapse dataset of the particular virus variant (WT, I18A, Y22A). The mask of ROIs was created based on the mCherry signal (mCherry signal-positive vesicles) and then the colocalization with the GagEGFP signal was quantified by using the Coloc2 analysis tool in ImageJ (version 1.52g, National Institutes of Health, Bethesda, MD, USA). 

### 2.7. Transmission Electron Microscopy (TEM) Analysis

Transmission electron microscopy (TEM) analysis was performed as described previously [22]. Briefly, COS-1 cells producing M-PMV were washed with PBS 48 h post-transfection and fixed with 2.5% glutaraldehyde in 0.1 M cacodylate buffer, pH 7.5 and then postfixed in 1% osmium tetroxide, dehydrated in ethanol. The material was embedded in Agar 100 epoxy resin. Ultrathin sections were contrasted with saturated uranyl acetate and analyzed with a JEOL JEM-1200EX microscope operating at 60 kV.

## 3. Results

### 3.1. mCherryTM Protein Synthesis, Processing and Incorporation into Viral Particles

The preparation of plasmid encoding the whole M-PMV genome where Env is tagged with the mCherry fluorescence protein in TM was described previously [11]. To investigate the mechanism of Env incorporation into the virus particle, we engineered the I18A and Y22A mutations into mCherryTM and compared their trafficking pathways with the wild type. COS-1 cells were transfected with plasmids harboring M-PMV proviral DNA with either untagged Env, WT mCherryTM, I18A mCherryTM, or Y22A mCherryTM. At 48 h post-transfection, the cells were metabolically labeled for 25 min and chased for 2 h, 4 h and 8 h. 

**Figure 1:**
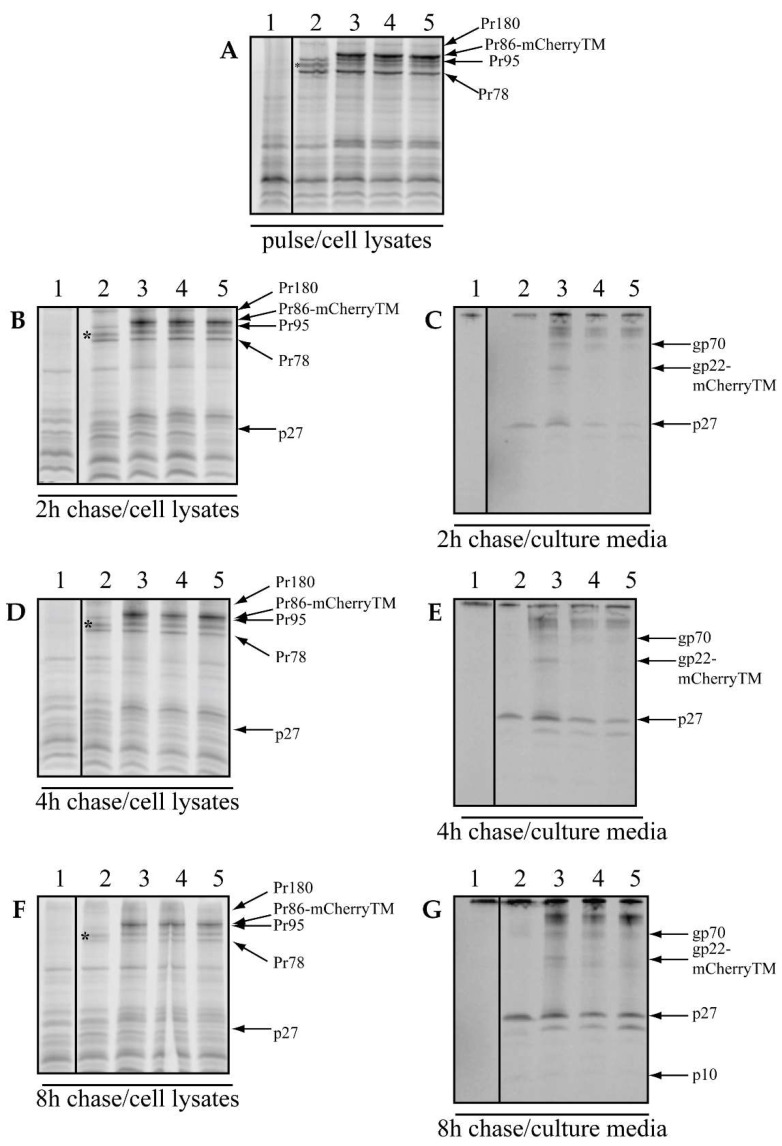
Sodium dodecyl sulfate polyacrylamide gel electrophoresis (SDS-PAGE) analysis of synthesis, processing and incorporation of wild-type (WT) and mutant Env with mCherry inserted into transmembrane domain (mCherryTM). COS-1 cells transfected with pSARMX-WT (untagged Env, lane 2), pSARMXmCherryTM WT (WT mCherryTM, lane 3), pSARMXmCherryTM I18A (I18A mCherryTM, lane 4) or pSARMXmCherryTM Y22A (Y22A mCherryTM, lane 5). Line 1 contains mock material corresponding to non-transfected cells. The cells were labeled 48 h posttransfection with Isolabel-[^35^S] for 25 min and chased in fresh culture media for 2 h, 4 h and 8 h. Viral proteins were immunoprecipitated with goat anti-Mason-Pfizer monkey virus (M-PMV) antibody and analyzed by SDS PAGE. The positions of the precursors Pr180 (Gag-Pro-Pol), Pr95 (Gag-Pro), Pr86 (Env-labeled *)), Pr78 (Gag), Pr86-mCherryTM; the product of the cleavage of Gag polyprotein, p27 (CA); product of the cleavage of Pr86, gp70 and product of the cleavage of Pr86-mCherryTM, gp22-mCherryTM are shown.

The molecular weights of the expressed mCherry-tagged Env precursors (Pr86-mCherryTM) corresponded well to that expected for fully glycosylated Env with mCherry inserted (~110 kDa) (Figure 1A, lanes 3–5). The signal intensities of these products are higher than for untagged Pr86 (Figure 1A, lane 2), due to the presence of 10 additional methionines in the mCherry tag. Importantly, the M-PMV structural and enzymatic polyprotein precursors Pr78 (Gag), Pr95 (Gag-Pro) and Pr180 (Gag-Pro-Pol) were expressed at similar levels for viruses expressing both untagged Env and mCherry-tagged Env (Figure 1A, lane 2 vs. lanes 3–5).

We observed a decrease in intracellular concentrations of the precursor proteins over the chase period (Figure 1B–F), which was accompanied by a reciprocal increase in signal present in the media corresponding to released virions (Figure 1C,E,G). This suggests that the precursors were efficiently incorporated and based on the presence of p27 (the Gag cleavage product originating from Pr180, Pr95 and Pr78 precursors that is linked to virus release), we conclude that virions underwent budding and the correct maturation process (Figure 1C,E,G; lines 2–5). The absence of gp22-mCherry in Figure 1C,E,G; lanes 4 and 5, is an expected result, since CT cleavage is mediated by the viral protease during maturation and neither I18A mCherryTM (line 4) nor Y22A mCherryTM (line 5) is expected to be incorporated into virions [9]. In contrast, the gp22-mCherry product was present in the culture medium collected from cells producing only the WT mCherryTM labeled virus (Figure 1C,E,G; lane 3). In both lanes 4 and 5 (Figure 1C,E,G), we also detected a protein consistent with gp70, a cleavage product of Pr86 and additional high-MW proteins likely released into the culture medium (Figure 1C,E,G; lanes 4 and 5). A western blot assay was performed to confirm the packaging of mCherryTM into virions by isolating and analyzing the proper density fractions of the iodixanol gradient to exclude any proteins which are not associated with the released particles. These experiments showed efficient incorporation of the mCherry-tagged gp22 and p27Gag into particles with the correct density for M-PMV virions (see Section SA in Supplementary Information). From these results, it is clear that WT mCherryTM was successfully transported to the plasma membrane for incorporation into viral particles and that the CT was properly processed during maturation, while this was not the case for viruses encoding the mCherry-tagged Env mutant proteins, consistent with previous work on Env lacking mCherry [9,14]. Moreover, the levels of p27 released into the culture medium were reduced for both the I18A and Y22A mutants, similar to the previous results of Song et al. [14]. 

### 3.2. The Intracellular Localization of mCherryTM and Its I18A and Y22A Mutants

Based on the above evidence that mCherryTM can serve as a relevant model for studying the trafficking of M-PMV Env, we sought to investigate its transport and localization in living cells under physiological conditions by fluorescence microscopy.

**Figure 2:**
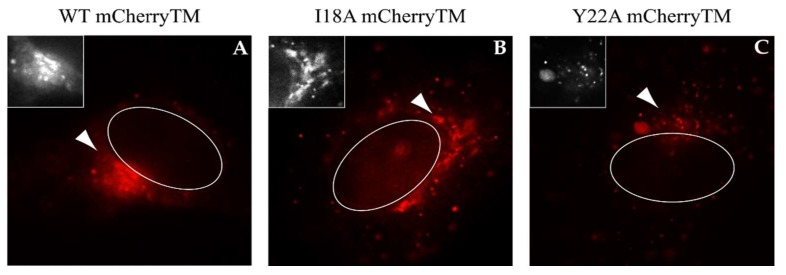
Intracellular localization of WT mCherryTM and I18A and Y22A mutants in living COS-1 cells. The COS-1 cells were transfected with pSARMXmCherryTM WT (**A**), pSARMXmCherryTM I18A (**B**) or pSARMXmCherryTM Y22A (**C**); 24 h posttransfection, they were imaged using the Olympus cell^R in real time. Each picture was captured as a single snap in the TxRed channel. All pictures were captured with the same exposure time. The magnified areas of each picture were transformed into a black-and-white version with increased contrast. The white ovals represent a nucleus. Magnification 600×; scale bar 20 µm.

**Figure 3:**
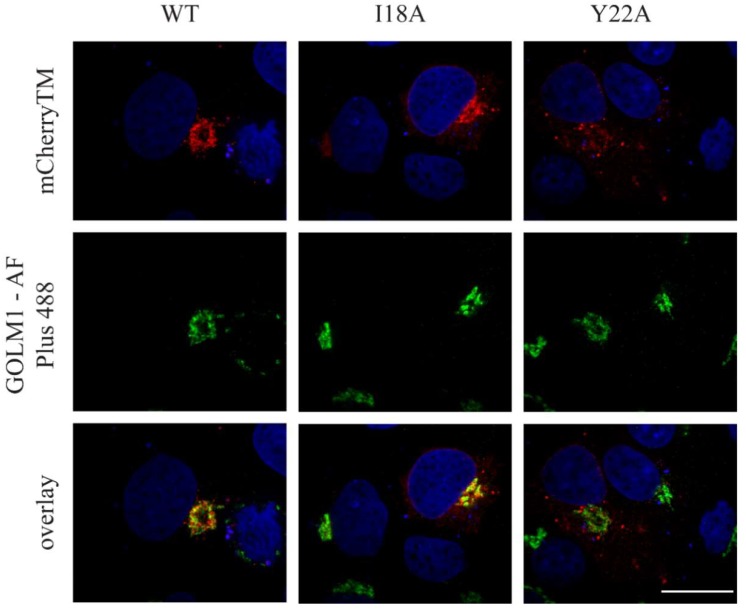
The colocalization of mCherryTM and cis/medial Golgi marker GOLM1. COS-1 cells were transfected with pSARMXmCherryTM WT or mutant variant. 48 h later, they were fixed with 4% formaldehyde and permeabilized with Tween 20. All samples were immunostained with primary rabbit anti-GOLM1 antibody and then with secondary antibody against rabbit IgG conjugated with Alexa Fluor ^TM^ Plus 488 and mounted into Vectashield mounting medium with DAPI. Samples were imaged with spinning disk confocal microscope (Andor). Magnification 600×; scale bar 20 µm.

As shown in Figure 2A–C, we did not observe any detectable mCherry signal at the plasma membrane; however, a strong mCherry signal was observed in the perinuclear region of the cells expressing WT mCherryTM, specifically, at cisternae-like organelles. Based on their shape and localization (Figure 2A), they are likely Golgi cisternae. Env is a type I transmembrane protein, thus the localization of mCherryTM to the Golgi would be expected, as these proteins are transported through the ER and Golgi for posttranslational processing (glycosylation, protease processing, etc.). In addition to the static region of intense staining with WT mCherryTM, we observed some signal in vesicles of unknown origin. Due to seeming variation among intracellular distribution of the signal for the Y22A mutant (Figure 2C), we performed additional experiments to verify whether the intensive mCherry signal localization in the perinuclear area is the Golgi apparatus. We immunostained cis/medial part of this organelle with anti-GOLM1 (Golgi membrane protein 1) antibody in cells producing WT mCherryTM or the mutant variants (Figure 3). The staining of the WT and the I18A mCherryTM mutant was equally intense, but I18A mCherryTM signal was somewhat more distributed around the GOLM1-labeled area. In contrast, the Y22A mCherryTM signal was detected in distributed cytoplasm vesicles, with a less intense signal colocalizing in the GOLM1-labeled region compared to the WT and the I18A mCherryTM mutant (Figure 3). 

### 3.3. Env and mCherryTM Localization at the Plasma Membrane (PM)

The lack of an mCherry signal at the plasma membrane for any of the mCherryTM constructs was quite surprising, as an intense signal of immunostained Env at the PM has previously been observed [9]. However, Song et al. analyzed cells producing Env in isolation, while we sought to detect Env in the presence of additional M-PMV proteins. To clarify, we replicated the antibody staining experiments performed by Song et al. using an M-PMV molecular clone (pSARMX-WT) or Env transport-defective I18A and Y22A variants. To directly replicate the observations made by Song et al., we also included pTMT-WT, which encodes M-PMV Env alone [14]. 

**Figure 4:**
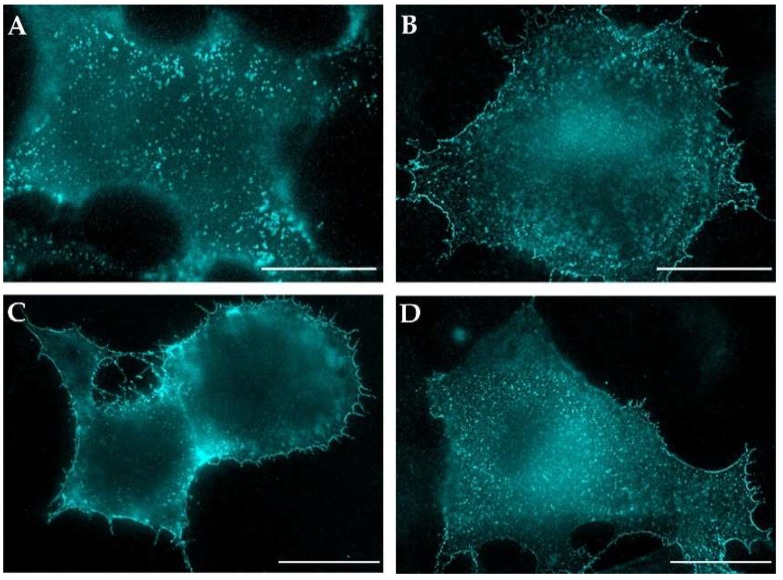
Plasma membrane distribution of M-PMV Env. The COS-1 cells were either transfected with plasmids encoding M-PMV genomic DNA with: WT Env (pSARMX-WT; panel **A**), I18A Env (pSARMX-I18A; panel **B**), or Y22A Env (pSARMX-Y22A; panel **C**) or an M-PMV Env expression vector (pTMT-WT; panel **D**). 48 h post-transfection cells were incubated with goat anti-M-PMV antibody on ice for 25 min to bind surface exposed M-PMV Env. Formaldehyde (4%) fixed cells were immunostained for CA protein with rabbit anti-CA antibody (except for the cells transfected with pTMT-WT,). Env was visualized using secondary anti-goat IgG antibody conjugated with Alexa Fluor^®^ 350. Samples were mounted in Vectashield mounting media and imaged on an Olympus cell^R microscope. The original blue color of the AF350 signal was changed to cyan for increased contrast. Magnification 600×; scale bars 20 µm.

Consistent with the findings of Song et al., we observed a largely uniform staining for M-PMV Env when expressed alone (Figure 4D). The Gag production in fixed and permeabilized cells was confirmed by counterstaining with the anti-CA antibody. 

Despite repeatedly observed somewhat larger speckles in panel A (Figure 4, complete virus) when compared to both mutant virus variant in panels B and C, we hesitate to speculate on relocalization of Env on the plasma membrane when Gag was co-expressed with either the wild type or the mutants, in contrast to that observed for HIV-1 [23]. The enhanced intensity of plasma membrane staining for the Y22A mutant (Figure 4C) indicates that its trafficking is retarded at the plasma membrane, presumably due to a defect in its endocytosis. This finding is in agreement with the observations of Song et al. for this mutant [9]. 

As was mentioned above, we failed to detect any mCherry-related fluorescence signal of the mCherryTM protein at the PM, however, since WT mCherryTM appeared to be incorporated into virions during budding, Env should be visible at the PM. To address this discrepancy between the missing signals of the mCherry-labeled Envs at PM and positive membrane signals of all non-mCherry-labeled Env proteins, we immunostained the surface of the COS-1 cells producing WT mCherryTM and its mutant variants.

**Figure 5:**
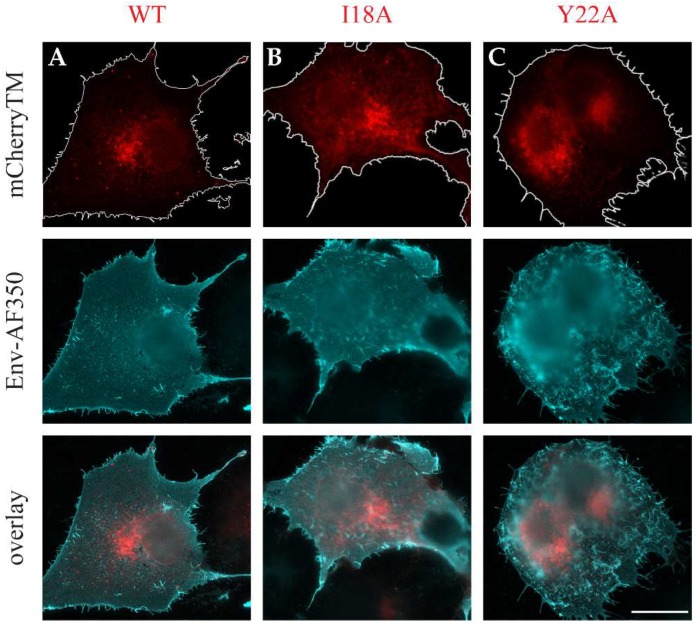
Immunostained mCherryTM proteins. COS-1 cells were transfected with plasmids encoding M-PMV genomic DNA with: mCherry-tagged Envs; pSARMXmCherryTM WT (**A**), pSARMXmCherryTM I18A (**B**) or pSARMXmCherryTM Y22A (**C**). After 48 h, the living cells were incubated with goat anti-M-PMV antibody on ice and then fixed with 4% formaldehyde and immunostained with secondary anti-goat IgG antibody conjugated with Alexa Fluor^®^ 350. The upper panels show mCherry fluorescence, the middle panels show the Alexa Fluor^®^ 350- staining, and the lower panels the two images overlayed. Non-transfected COS-1 cells were processed in the same set of samples, no non-specific staining was observed for this immunostaining (). The original blue color of AF350 signal was changed to cyan for increased contrast. The delineation in the upper panels highlights the plasma membrane (PM). Magnification 600×; scale bar 20 µm.

The non-permeabilized cells expressing M-PMV with mCherry-labeled Env proteins produced intense intracellular mCherry signals, but little or no evidence of an mCherry signal at the plasma membrane. In contrast, the same cells immunostained with anti-M-PMV antibody exhibited intense PM staining, with the signal homogenously distributed over the surface of the cell (Figure 5A–C). It therefore appears that both the WT Env (Figure 4) and WT mCherryTM protein is present at the same levels at the cell surface. The lack of the mCherry signal at the PM remains to be explained. The explanation could be either its presence at low concentration that is under the detection limit of the inherent mCherry signal or a loss of its ability to fluoresce following exposure to the culture medium. 

**Figure 6:**
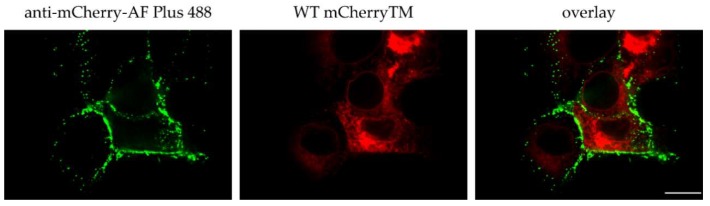
Membrane localization of WT mCherryTM immunostained with anti-mCherry antibody. COS-1 cells were transfected with pSARMXmCherryTM WT. After 48 h, the living cells were incubated with rabbit anti-mCherry antibody on ice and then fixed with 4% formaldehyde and immunostained with secondary anti-rabbit IgG antibody conjugated with Alexa Fluor^TM^ Plus 488. The left panel shows surface immune-staining of mCherry, the middle panel shows the inherent mCherry fluorescence, and the right panel shows these two images overlayed. Mock-transfected COS-1 cells were processed in the same way; no signal was detected. One focal plane acquired with a spinning disk confocal microscope (Andor) is showed. Magnification 600×; scale bar 20 µm.

To address this question, we performed surface staining of intact non-permeabilized cells using anti-mCherry antibody labeled with the high signal-to-noise ratio fluorophore Alexa Fluor^TM^ Plus 488. The immunofluorescence image (Figure 6, the right panel) exhibits an intensive green signal corresponding to the presence of mCherryTM at the membrane surface. As seen from the middle panel in Figure 6, the intracellular mCherry signal is substantially higher than the very faint, close to zero, mCherry signal at the membrane. Nevertheless, these experiments show that the mCherryTM is efficiently transported to the plasma membrane and argue that this protein can be employed to study the intracellular trafficking of Env.

### 3.4. Characterization of Vesicles Carrying Mason-Pfizer Monkey Virus (M-PMV) Env

**Figure 7:**
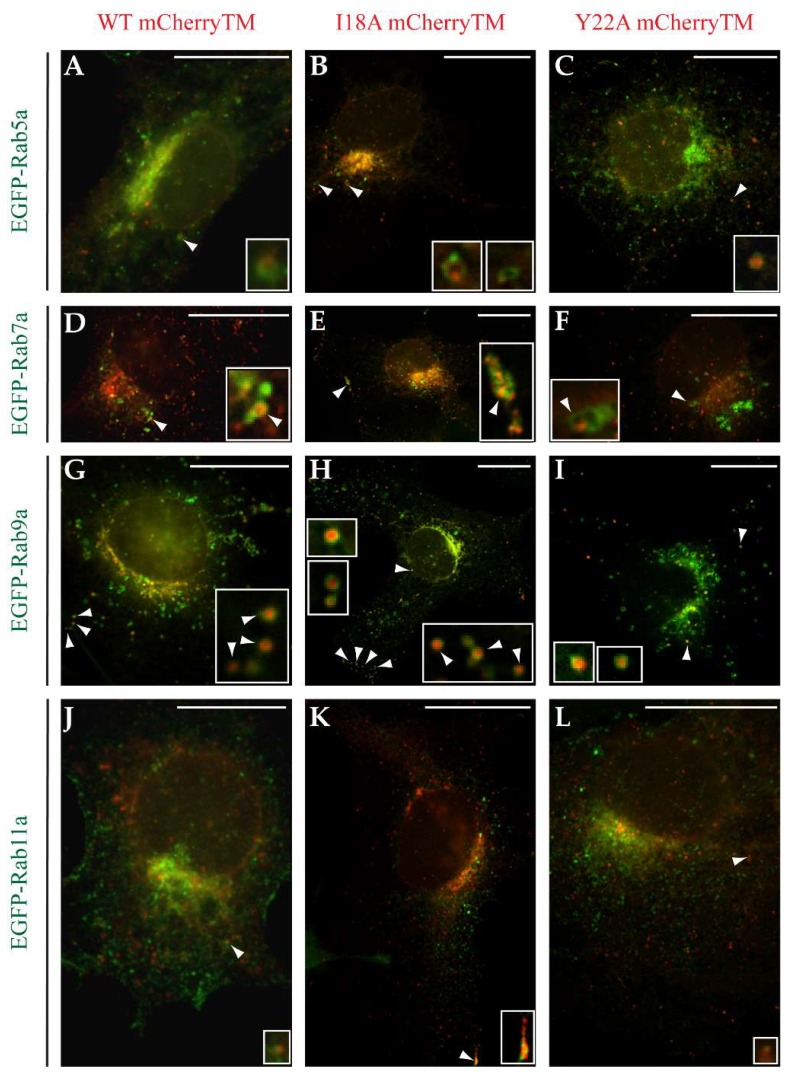
Rab-markers based identification of intracellular vesicles carrying mCherryTM protein variants. The COS-1 cells co-transfected with a combination of pSARMXmCherryTM and enhanced green fluorescent protein (EGFP)-tagged endosomal marker coding plasmids were fixed with 4% formaldehyde 24 h post-transfection. Upon mounting in Vectashield media the samples were imaged using an Olympus cell^R microscope. White arrowheads indicate colocalization of the mCherryTM signal and the corresponding marker. Magnification 600×; scale bars 20 µm.

Based on the shape, size, and mobility in live cell imaging of the Env-containing vesicles, along with previous observations that the M-PMV Env cytoplasmic tail targets viral glycoproteins to endosomal pathways [24], we concluded that the pool of mobile mCherryTM is present in the form of endosomal vesicles. In order to explore this, Rab5a, Rab7a, Rab9a, and Rab11a, four markers of specific endosomal compartments N-terminally tagged with EGFP were co-expressed with WT or mutant mCherryTM (Figure 7). All the markers belong to the family of the Ras superfamily of G proteins (Rab GTPases) and are responsible for regulating the transport and maturation of endosomal vesicles. Rab5a regulates transport from the plasma membrane to the early endosomes [25] and is predominantly localized to the early/sorting endosomes. Rab7a moderates the maturation of certain domains of early/sorting endosomes into late endosomes [26], and so Rab7a is frequently used as a marker of late endosomes. Rab9a is involved in the retrograde transport of cargo from late endosomes to the TGN [27]. Rab11a specifically localizes to recycling endosomes, which recycle cargo from early/sorting endosomes back to the plasma membrane [10,28]. 

We did not observe any significant colocalization of WT mCherryTM with Rab5a-positive early/sorting endosomes (Figure 7A). Only approximately 3% of all identified Rab5-positive vesicles also contained WT mCherryTM signal. While surprising, since previous work by Song showed the rapid endocytosis of WT Env, this could have resulted from the reduced mCherryTM signal observed following its exposure on the PM. Also, approximately 7% of Rab5a-positive vesicles colocalized with the I18A mCherryTM signal (Figure 7B). As shown in the magnified field in Figure 7B, while the I18A mCherryTM and EGFP-tagged Rab5a marker were present on the same vesicles, they did not colocalize in particular subdomains. 

The Y22A mutant disrupts a critical endocytic signal, and results in a significant deficiency in its removal from the plasma membrane [9]. Based on this, it was surprising that colocalization between Y22A mCherryTM and Rab5a was identified (Figure 7C). Based on the analysis, 10% of Rab5-positive vesicles contained also Y22A mCherryTM signal. 

In contrast to what was observed for Rab5a, all three mCherryTM fusion proteins significantly colocalized with Rab7a. The highest degree of colocalization with Rab7a was seen for the WT Env, where 68% of Rab7a-positive vesicles also contained the WT mCherryTM signal (Figure 7D), while both the I18A (Figure 7E) and Y22A (Figure 7F) mutants colocalized with Rab7a much less efficiently (23% for I18A variant and 9% for Y22A variant). The colocalization between mCherryTM and Rab9a-EGFP-positive endosomes was found to be 30% for WT mCherryTM, 23% for I18A mCherryTM and 25% for Y22A mCherryTM (Figure 7G–I). This confirmed a previous report that Env CT directs localization to Rab9a-positive endosomes [24], and shows that the observation applies both in the context of full-length Env and in the presence of other viral proteins. Only a minor degree of colocalization was detected for mCherryTM variants on Rab11a-positive recycling endosomes, where only about 2% of recycling endosomes contained a WT mCherryTM signal, and about 1% of such vesicles contained an I18A mCherryTM and an Y22A mCherryTM signal. (Figure 7J–L). 

### 3.5. Live-Cell Imaging of mCherryTM and EGFP-Tagged M-PMV Virus Like Particle

pSARM-Gag-EGFP-M100A was used to monitor interactions between M-PMV Gag and mCherryTM and the trafficking properties of Gag-associated Env vesicles. This vector has been previously described; but briefly, it expresses a Gag C-terminally tagged with EGFP, does not encode Pro or Pol, and Env production is significantly decreased due to the effect of codon optimization of the Gag sequence [12]. COS-1 cells were co-transfected with pSARM-Gag-EGFP-M100A and WT or mutant mCherryTM and 24 h post-transfection samples were imaged with an Olympus cell^R microscope under physiological conditions in real time.

**Figure 8:**
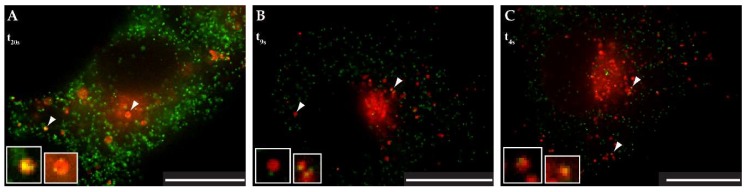
Localization of mCherryTM protein variants and EGFP-labeled Gag/virus like particles. The COS-1 cells were seeded into glass-bottomed dishes and co-transfected 24 h later with pSARMXmCherryTM WT (panel **A**) or one of the mutant variants (pSARMXmCherryTM I18A, panel **B**; pSARMXmCherryTM Y22A, panel **C**) together with the pSARM-Gag-EGFP-M100A constructs. The next day, the living cells were imaged in the Olympus cell^R microscope under physiological conditions. Panel **A** shows WT mCherryTM and EGFP-labeled immature capsids (see also Videos S1–S4 in Supplementary Information). Panel **B** shows I18A mCherryTM and EGFP-labeled immature capsids (see Video S5 in Supplementary Information). Panel **C** shows Y22A mCherryTM and EGFP labeled immature capsids (see Video S6 in Supplementary Information). White arrowheads indicate mCherryTM-containing vesicles colocalizing with Gag-EGFP virus-like particles. Magnification 600×; scale bar 20 µm.

Figure 8A shows one time point (t20s) from videos of WT mCherryTM and pSARM-Gag-EGFP-M100A co-transfected COS-1 cells (Video S1 and S2, magnified region in Video S3 and S4). The white arrowhead in this figure highlights a vesicle carrying at least two immature capsids, and in the associated video it is clear that this vesicle is being trafficked towards the plasma membrane. Another 8–13 WT mCherryTM-containing vesicles with attached immature capsids near the PM can be seen and at least three more are visible in the perinuclear area. The colocalization of WT mCherryTM with GagEGFPWT was quantified with Coloc2 tool in ImageJ (version 1.52g, National Institutes of Health, Bethesda, MD, USA). A rounded estimate is that 47% of identified WT mCherryTM-positive vesicles colocalized with the GagEGFP signal. It needs to be emphasized that this particular dataset was challenging for software-based analysis, due to the very high density of GagEGFP-positive particles, resulting in a high background signal which had an impact on the measured parameters. This was not the case for both mutant variants. However, by visual analysis, we estimated that up to 73% of WT mCherryTM vesicles colocalized with GagEGFP particles.

With I18A, the software analysis was more accurate according to the almost zero background signal. 

The colocalization rate of I18A mCherryTM and Gag-EGFP (white arrowheads in Figure 8B, shown one time point-t9s) was 33% which is significantly lower than for WT Env. This is consistent with the reduced incorporation of this mutant Env. I18A mCherryTM vesicles oscillating near the nucleus were observed (Video S5). The data documenting the vesicular co-transport of Y22A mCherryTM and GagEGFP immature capsids (Figure 8C, shown one time point-t4s) were similar to that of I18A mCherryTM (Video S6), even in that the colocalization rate was 53%, which is almost halfway between WT and I18A Env. As was observed for the I18A mutant, a majority of the vesicles associated with immature capsids were near the nucleus. To analyze the type of transport of a particular mCherry variant, particle tracking measurements were performed (see Section SB in Supplementary Information). 

**Figure 9:**
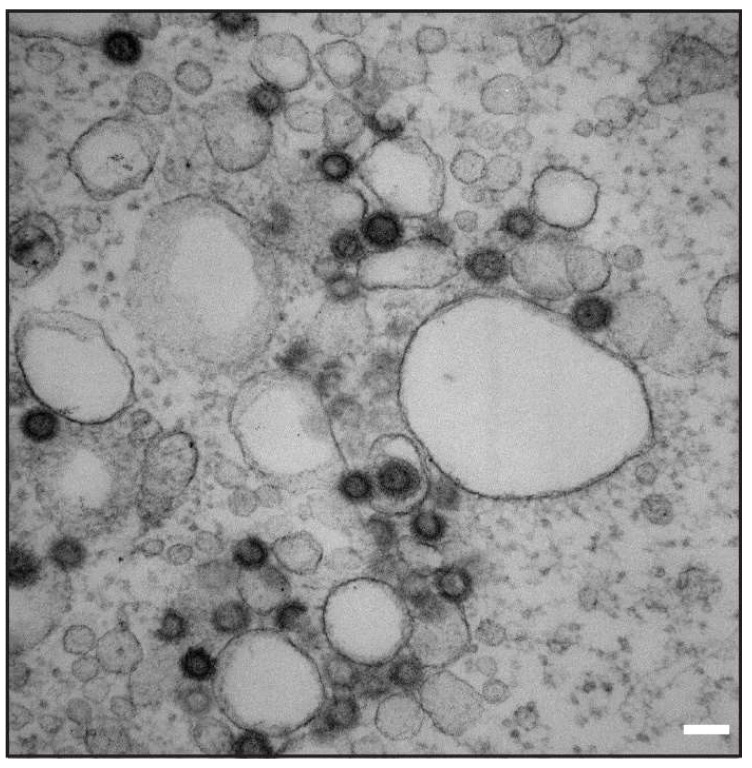
Transmission electron microscope (TEM) image of immature M-PMV particles associated with vesicular membranes. Scale bar represents 100 nm.

The association of WT immature particles with intracellular vesicles was also confirmed by the transmission electron microscopy of COS-1 cells transfected with whole M-PMV provirus vector (Figure 9). 

## 4. Discussion

Several studies focusing on the molecular mechanism of M-PMV Env intracellular trafficking and its interaction with Gag have been published [5,10,11]. The main focus of these studies was on the role of the CT since it is the only part of Env available for potential interactions with viral structural polyproteins and cytoplasmic proteins. It is important in the initiation of Env incorporation into the viral particle, and it also plays a role in anterograde and retrograde trafficking, and in the initiation of intracellular signaling cascades leading to enhanced viral gene expression [29,30,31,32].

In M-PMV, the CT is only 38 amino acids long but it contains at least two signal sequences that are important for the regulation of intracellular transport and interaction of Env with Gag [33]. Alanine-scanning mutagenesis identified three residues critical for Env incorporation into budding virions. Mutations I18A, Y22A completely disrupt incorporation and L25A mutant is poorly incorporated [9,14]. Intracellularly assembled M-PMV immature capsids acquire their Env at the plasma membrane during budding [1,34,35], however it is still unclear where the initial contact between Env and Gag occurs and which domain of Gag is responsible for this interaction. Based on observations in HIV-1, where a single mutation in MA or deletion in the CT blocks Env incorporation, M-PMV MA is presumed to mediate this interaction [32,36,37,38]. Using live-cell imaging of fluorescently tagged Gag and Env, we determined dynamic intracellular interactions between Gag immature capsids and Env-containing vesicles (Figure 8A–C and Videos S1–S6). This system enables the intracellular trafficking of M-PMV Env to be tracked. The particle-tracking analysis showed that all three variants are transported via active transport (see Section SB in Supplementary Information).

Env, as a type I transmembrane protein is synthesized at the rough ER and it is glycosylated and cleaved by furin while traversing toward the *trans*-Golgi apparatus (TGN). Once Env reaches the TGN, it must be sorted to either cisterna C6 or C7 of TGN. The cargo leaving the C6 cisterna is transported to the plasma membrane via long tubules and nonclathrin buds, while cargo leaving the C7 cisterna is targeted exclusively to the endosomal pathway via clathrin-coated vesicles [39,40]. It is unclear how the sorting of transmembrane proteins into these cisternae occurs; however, the length of the membrane-spanning domain (MSD) likely plays a role in this process. It was originally shown for sorting at early endosomes that extension of the MSD of TGN38 from 21 to 24 amino acids caused an aberrant localization of proteins into distinct microdomains [41]. In addition, White et al. demonstrated that the length of the MSD together with signals in the cytoplasmic domain assist in segregating transmembrane proteins from Golgi resident proteins into distinct “cargo” regions [42]. The M-PMV MSD consists of 28 amino acids [33] and could be the factor influencing Env targeting into the distinct cisterna of the TGN. 

In contrast to the C-type retroviruses, M-PMV particles assemble at the centrioles, and Env was thought to be transported from the TGN directly to the plasma membrane [9,43]. Here the endocytic signal present in the CT mediates rapid Env endocytosis. This generally prevents prolonged exposure to the immune system. Sfakianos et al. proposed that M-PMV particles assemble at the membrane of recycling endosomes [10]. This could subsequently result in the efficient targeting of both Env and Gag. Evidence for an interaction between M-PMV immature capsids and intracellular vesicles being utilized in anterograde transport has been published by our group previously [5,11]. This intracellular interaction is also consistent with the finding that M-PMV MA interacts with the plasma membrane phospholipids with significantly lower affinity [44] compared to HIV-1. We also observed an accumulation of Gag at intracellular vesicles in COS-1 cells producing wild-type untagged M-PMV soon after infection and also transmission electron microscopy has confirmed the presence of assembled M-PMV particles associated with intracellular vesicles (Figure 9).

Using the plasmid pSARMXmCherryTM encoding whole M-PMV with mCherry tagged Env, we observed Env in living cells. The majority of mCherryTM was present in intracellular vesicles and cis/medial Golgi (Figure 2 and Figure 3). The predominant vesicular localization supports the theory that a significant fraction of Env is targeted to the C7 cisterna of TGN and then to the endosomal pathway rather than to C6 cisterna and the PM. Blot et al. published a similar observation utilizing M-PMV Env CT fused to the C terminus of the CD25 protein. They proposed that this chimera is targeted directly from the C7 cisterna to the Rab7a-positive endosomes and then (based on the length of MSD and targeting of Env into distinct microdomains on endosomal membranes) to the Rab9a-positive endosomes [24], where Rab9aGTPase moderates the recycling of cargo back to the TGN [27,45]. They suggested that Env is retained in this intracellular cycle (TGN-Endosomal pathway-TGN) to avoid its unnecessary exposure on the PM [24]. Our data are in agreement with this theory of intracytoplasmic Env recycling. All three tested mCherry tagged M-PMV variants exhibited strong colocalization with the Rab7a marker and moderate colocalization with the Rab9a marker (see Figure 7D–I). We observed only a low colocalization of all three variants with the Rab5a marker of early endosomes (Figure 7A–C), consistent with the majority of Env entering the endosomal pathway directly from the TGN, although this could reflect the much reduced mCherry signal observed at the plasma membrane and possible quenching of the signal following extracellular exposure. 

Blot et al. showed that the dileucine motif (LL or LΦ motif) mediates the targeting of M-PMV Env CT containing the chimera from the TGN to late endosomes, while a tyrosine-based motif (the YxxΦ motif) mediates retrograde transport from Rab9a-positive endosomes to the TGN [24]. Our data confirm the importance of the YxxΦ motif in Env transport, as we used M-PMV Env with its native trimerization domain and complete SU and TM subunits. As shown in Figure 2C and Figure 3, the Y22A mCherryTM protein localizes predominantly in endosomal vesicles while the WT and I18A (Figure 2A,B and Figure 3) variants are concentrated more also on cisterna-like organelles, labeled with GOLM1 (cis/medial Golgi) marker, suggesting that the Y22A mutation blocks recycling from Rab9a vesicles to the TGN. 

In the cells producing only Env (without other viral proteins), we observed an intense signal of immunostained Env present on the PM (Figure 4D), consistent with the observations of Song et al. [9,14]. We saw a similar result in cells producing both complete untagged WT and also mutated viruses (Figure 4A–C) and a mCherry-tagged virus-infected cell stained with anti-mCherry antibody (Figure 6), where Gag is present. This Env-related signal on plasma membrane is either highlighting spots where budding of virus particles occurs or Env that has reached the plasma membrane but failed to be incorporated to the virus.

Therefore, we hypothesize that Env can follow two intracellular routes following its synthesis; in the first, Env cycles internally via Rab9 vesicles to interact with immature capsids just assembling in the pericentriolar region, and in the second, Env is transported directly to the PM.

**Figure 10:**
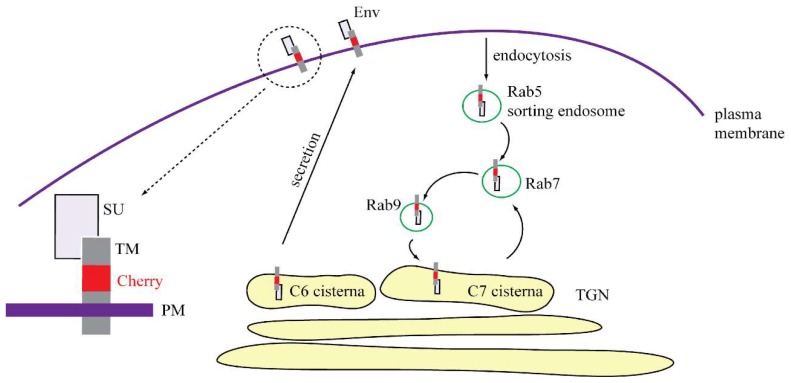
Proposed scheme of M-PMV Env intracellular transport when expressed in the absence of other viral components.

Based on our findings together with previously published data, we propose the mechanism depicted in Figure 10, where two subpopulations of Env leave the TGN. The first is directly transported to the PM and, because the endocytic signal is not hindered by interaction with immature capsids, Env is rapidly endocytosed and thus targeted to Rab5a-positive endosomes. Based on MSD properties and signals in the CT, Env is present in distinct microdomains at the membrane of this endosome, which matures into a Rab7a-positive endosome. Such, Rab7a-positive endosomes have been shown to recruit Rab9aGTPase into distinct microdomains [26,41,46,47], which afterwards mature into Rab9a-positive endosomes recycling cargo from late endosomes to cisterna C7 of the TGN [48]. The Rab9aGTPase remains connected with these vesicles until their fusion with the TGN membrane [27]. At least some of these Env molecules could then be transported from the TGN, likely utilizing a dileucine motif in the CT back to Rab7a endosomes. This motif was shown to interact with adaptor proteins to recruit clathrin to the site of bud formation [49,50].

The existence of two independent pathways might also explain published observations that disruption of the dileucine motif did not prevent the incorporation of Env into M-PMV virions [14]. The disruption of the dileucine motif would block the targeting of Env from the TGN to the endosomal pathway but would not prevent Env transport to the PM and subsequent endocytosis back to Rab7 endosomes. In contrast, disruption of the YxxΦ motif may stop both routes at the membrane of Rab9a-positive vesicles. Since we have demonstrated the co-transport of Y22A mCherryTM with the immature capsid (Figure 8, Video S6), the Y22A mutation probably did not influence the recruitment of immature capsids to the endosomal membrane or their anchoring to the endosomal membrane. Nevertheless, it presumably prevents the recycling of Env from Rab9a endosomes back to the TGN and then transport to the PM. This would explain the lower proportion of the Y22A mutant signal in the GOLM1 labeled region, where both the WT and I18A mutant are predominantly observed (Figure 3).

**Figure 11:**
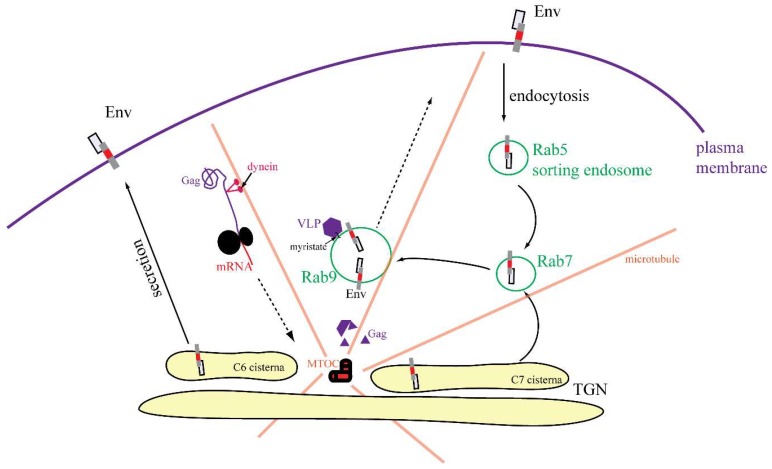
Proposed scheme of M-PMV Env and Gag intracellular transport.

We propose a model of Env transport in the M-PMV-infected cell (Figure 11), where Gag that is transported to the MTOC via interaction with a light chain of dynein (Tctex-1) [4] oligomerizes in this region into the hexameric lattice of the immature particle. These preassembled particles can bind to the adjacent Env-containing Rab9a-positive vesicles either directly through interaction with polar lipids [51] or via the cytoplasmic tail of the TM. The transport of Rab9a-positive endosomes towards both the plasma membrane and the TGN has been shown by Barbero et al. [27]. We hypothesize that the interaction of immature capsids with the Env-enriched vesicles can induce a switch in transport towards the PM. This is supported by live-cell microscopy, where we observed immature capsid-associated-Env-carrying vesicles near the nucleus as well as on the cell periphery (Figure 8, Video S1–S6).

In summary, by using biochemical methods, fluorescence microscopy including live-cell imaging, and TEM, we analyzed the intracellular trafficking of M-PMV Gag and Env and propose a scheme of intracellular trafficking of these viral components via the endocytic sorting machinery (Figure 11). The data extend and refine those published by Sfakianos et al. and Blot et al., who suggested that Env and Gag traverse jointly through the cytoplasm of infected cells [10,24].

## Supplementary Materials

The following are available online at http://www.mdpi.com/1999-4915/10/10/575/s1, Video S1 and S2: Live imaging of COS-1 cell co-transfected with pSARMXmCherryTM WT and pSARM-Gag-EGFP-M100A to demonstrate co-transport of EGFP-tagged M-PMV capsids and WT mCherryTM-containing vesicle. Focused on the whole cell. Fast version means compression of 10 frames per second and shows real velocity of movement (Video S1). Slow version means compression of five frames per second (Video S2). Video S3 and S4: Live imaging of COS-1 cell co-transfected with pSARMXmCherryTM WT and pSARM-Gag-EGFP-M100A to demonstrate co-transport of EGFP-tagged M-PMV capsids and WT mCherryTM-containing vesicle. Focused on small region on periphery of cell. Fast version means compression of 10 frames per second and shows real velocity of movement (Video S3). Slow version means compression of five frames per second (Video S4). Video S5: Live imaging of COS-1 cell co-transfected with pSARMXmCherryTM I18A and pSARM-Gag-EGFP-M100A to demonstrate co-transport of EGFP-tagged M-PMV capsid and I18A mCherryTM-containing vesicle. Focused on the whole cell. Only one velocity provided. Video S6: Live imaging of COS-1 cell co-transfected with pSARMXmCherryTM Y22A and pSARM-Gag-EGFP-M100A to demonstrate co-transport of EGFP-tagged M-PMV capsid and Y22A mCherryTM-containing vesicle. Focused on the whole cell. Only one velocity provided. Section SA—Western blot analysis of released virions. Released virions were isolated via iodixanol gradient. Only the fractions of the proper density, corresponding to the density of the virions [52], were analyzed. Antibody against Gag and against mCherry were used to visualize particular viral components. Presence of gp22mCherry signal in isolated virions was validated. Section SB—Particle-tracking analysis was performed using Fiji plugin Mosaic: Particle tracker 2D/3D [53,54]. Two cells for each mCherry virus variant from independent experiments were selected for this analysis. In all analyzed cells, 6 to 8 mCherry signal-containing vesicles were tracked. The trajectories were filtered to only include those containing at least 50 time points. The analysis showed, according to the MSS slope values, that all three variants were using active transport.

## References

[1] Chopra H.C., Mason M.M. (1970). A new virus in a spontaneous mammary tumor of a rhesus monkey. Cancer Res..

[2] Kramarsky B., Sarkar N.H., Moore D.H. (1971). Ultrastructural comparison of a virus from a Rhesus-monkey mammary carcinoma with four oncogenic RNA viruses. Proc. Natl. Acad. Sci. USA.

[3] Kohoutova Z., Rumlova M., Andreansky M., Sakalian M., Hunter E., Pichova I., Ruml T. (2009). The impact of altered polyprotein ratios on the assembly and infectivity of Mason-Pfizer monkey virus. Virology.

[4] Vlach J., Lipov J., Rumlova M., Veverka V., Lang J., Srb P., Knejzlik Z., Pichova I., Hunter E., Hrabal R. (2008). D-retrovirus morphogenetic switch driven by the targeting signal accessibility to Tctex-1 of dynein. Proc. Natl. Acad. Sci. USA.

[5] Sfakianos J.N., LaCasse R.A., Hunter E. (2003). The M-PMV cytoplasmic targeting-retention signal directs nascent Gag polypeptides to a pericentriolar region of the cell. Traffic.

[6] Sakalian M., Hunter E. (1999). Separate assembly and transport domains within the Gag precursor of Mason-Pfizer monkey virus. J. Virol..

[7] Hong S., Choi G., Park S., Chung A.S., Hunter E., Rhee S.S. (2001). Type D retrovirus Gag polyprotein interacts with the cytosolic chaperonin TRiC. J. Virol..

[8] Ulbrich P., Haubova S., Nermut M.V., Hunter E., Rumlova M., Ruml T. (2006). Distinct roles for nucleic acid in in vitro assembly of purified Mason-Pfizer monkey virus CANC proteins. J. Virol..

[9] Song C., Dubay S.R., Hunter E. (2003). A Tyrosine Motif in the Cytoplasmic Domain of Mason-Pfizer Monkey Virus Is Essential for the Incorporation of Glycoprotein into Virions. J. Virol..

[10] Sfakianos J.N., Hunter E. (2003). M-PMV capsid transport is mediated by Env/Gag interactions at the pericentriolar recycling endosome. Traffic.

[11] Pereira L.E., Clark J., Grznarova P., Wen X., Lacasse R., Ruml T., Spearman P., Hunter E. (2014). Direct evidence for intracellular anterograde co-transport of M-PMV Gag and Env on microtubules. Virology.

[12] Clark J., Grznarova P., Stansell E., Diehl W., Lipov J., Spearman P., Ruml T., Hunter E. (2013). A Mason-Pfizer Monkey Virus Gag-GFP Fusion Vector Allows Visualization of Capsid Transport in Live Cells and Demonstrates a Role for Microtubules. PLoS ONE.

[13] Choudhury A., Dominguez M., Puri V., Sharma D.K., Narita K., Wheatley C.W., Marks D.L., Pagano R.E. (2002). Rab proteins mediate Golgi transport of caveola-internalized glycosphingolipids and correct lipid trafficking in Niemann-Pick C cells. J. Clin. Investig..

[14] Song C., Micoli K., Bauerova H., Pichova I., Hunter E. (2005). Amino acid residues in the cytoplasmic domain of the Mason-Pfizer monkey virus glycoprotein critical for its incorporation into virions. J. Virol..

[15] Rumlova M., Ruml T., Pohl J., Pichova I. (2003). Specific in vitro cleavage of Mason-Pfizer monkey virus capsid protein: Evidence for a potential role of retroviral protease in early stages of infection. Virology.

[16] Schindelin J., Arganda-Carreras I., Frise E., Kaynig V., Longair M., Pietzsch T., Preibisch S., Rueden C., Saalfeld S., Schmid B. (2012). Fiji: An open-source platform for biological-image analysis. Nat. Methods.

[17] Schindelin J., Rueden C.T., Hiner M.C., Eliceiri K.W. (2015). The ImageJ ecosystem: An open platform for biomedical image analysis. Mol. Reprod. Dev..

[18] Schneider C.A., Rasband W.S., Eliceiri K.W. (2012). NIH Image to ImageJ: 25 years of image analysis. Nat. Methods.

[19] Costes S.V., Daelemans D., Cho E.H., Dobbin Z., Pavlakis G., Lockett S. (2004). Automatic and quantitative measurement of protein-protein colocalization in live cells. Biophys. J..

[20] Manders E.M.M., Verbeek F.J., Aten J.A. (1993). Measurement of co-localization of objects in dual-colour confocal images. J. Microsc..

[21] Li Q., Lau A., Morris T.J., Guo L., Fordyce C.B., Stanley E.F. (2004). A syntaxin 1, Galpha(o), and N-type calcium channel complex at a presynaptic nerve terminal: Analysis by quantitative immunocolocalization. J. Neurosci. Off. J. Soc. Neurosci..

[22] Bohmova K., Hadravova R., Stokrova J., Tuma R., Ruml T., Pichova I., Rumlova M. (2010). Effect of dimerizing domains and basic residues on in vitro and in vivo assembly of Mason-Pfizer monkey virus and human immunodeficiency virus. J. Virol..

[23] Muranyi W., Malkusch S., Muller B., Heilemann M., Krausslich H.G. (2013). Super-resolution microscopy reveals specific recruitment of HIV-1 envelope proteins to viral assembly sites dependent on the envelope C-terminal tail. PLoS Pathog..

[24] Blot V., Lopez-Verges S., Breton M., Pique C., Berlioz-Torrent C., Grange M.P. (2006). The conserved dileucine- and tyrosine-based motifs in MLV and MPMV envelope glycoproteins are both important to regulate a common Env intracellular trafficking. Retrovirology.

[25] Bucci C., Parton R.G., Mather I.H., Stunnenberg H., Simons K., Hoflack B., Zerial M. (1992). The small GTPase rab5 functions as a regulatory factor in the early endocytic pathway. Cell.

[26] Vanlandingham P.A., Ceresa B.P. (2009). Rab7 regulates late endocytic trafficking downstream of multivesicular body biogenesis and cargo sequestration. J. Boil. Chem..

[27] Barbero P., Bittova L., Pfeffer S.R. (2002). Visualization of Rab9-mediated vesicle transport from endosomes to the trans-Golgi in living cells. J. Cell Boil..

[28] Ullrich O., Reinsch S., Urbe S., Zerial M., Parton R.G. (1996). Rab11 regulates recycling through the pericentriolar recycling endosome. J. Cell Boil..

[29] Postler T.S., Bixby J.G., Desrosiers R.C., Yuste E. (2014). Systematic analysis of intracellular trafficking motifs located within the cytoplasmic domain of simian immunodeficiency virus glycoprotein gp41. PLoS ONE.

[30] Postler T.S., Desrosiers R.C. (2013). The tale of the long tail: The cytoplasmic domain of HIV-1 gp41. J. Virol..

[31] Postler T.S., Desrosiers R.C. (2012). The cytoplasmic domain of the HIV-1 glycoprotein gp41 induces NF-kappaB activation through TGF-beta-activated kinase 1. Cell Host Microbe.

[32] Tedbury P.R., Freed E.O. (2015). The cytoplasmic tail of retroviral envelope glycoproteins. Prog. Mol. Boil. Transl. Sci..

[33] Sonigo P., Barker C., Hunter E., Wain-Hobson S. (1986). Nucleotide sequence of Mason-Pfizer monkey virus: An immunosuppressive D-type retrovirus. Cell.

[34] Rhee S.S., Hunter E. (1991). Amino-Acid Substitutions within the Matrix Protein of Type-D Retroviruses Affect Assembly, Transport and Membrane Association of a Capsid. EMBO J..

[35] Rhee S.S., Hunter E. (1987). Myristylation Is Required for Intracellular-Transport but Not for Assembly of D-Type Retrovirus Capsids. J. Virol..

[36] Murakami T., Freed E.O. (2000). Genetic evidence for an interaction between human immunodeficiency virus type 1 matrix and alpha-helix 2 of the gp41 cytoplasmic tail. J. Virol..

[37] Freed E.O., Martin M.A. (1995). Virion incorporation of envelope glycoproteins with long but not short cytoplasmic tails is blocked by specific, single amino acid substitutions in the human immunodeficiency virus type 1 matrix. J. Virol..

[38] Mammano F., Kondo E., Sodroski J., Bukovsky A., Gottlinger H.G. (1995). Rescue of human immunodeficiency virus type 1 matrix protein mutants by envelope glycoproteins with short cytoplasmic domains. J. Virol..

[39] Ladinsky M.S., Kremer J.R., Furcinitti P.S., McIntosh J.R., Howell K.E. (1994). HVEM tomography of the trans-Golgi network: Structural insights and identification of a lace-like vesicle coat. J. Cell Boil..

[40] Ladinsky M.S., Mastronarde D.N., McIntosh J.R., Howell K.E., Staehelin L.A. (1999). Golgi structure in three dimensions: Functional insights from the normal rat kidney cell. J. Cell Boil..

[41] Chia P.Z., Gasnereau I., Lieu Z.Z., Gleeson P.A. (2011). Rab9-dependent retrograde transport and endosomal sorting of the endopeptidase furin. J. Cell Sci..

[42] White J., Keller P., Stelzer E.H. (2001). Spatial partitioning of secretory cargo from Golgi resident proteins in live cells. BMC Cell Boil..

[43] Hunter E., Swanstrom R. (1990). Retrovirus envelope glycoproteins. Curr. Top. Microbiol. Immunol..

[44] Prchal J., Srb P., Hunter E., Ruml T., Hrabal R. (2012). The structure of myristoylated Mason-Pfizer monkey virus matrix protein and the role of phosphatidylinositol-(4,5)-bisphosphate in its membrane binding. J. Mol. Boil..

[45] Lombardi D., Soldati T., Riederer M.A., Goda Y., Zerial M., Pfeffer S.R. (1993). Rab9 functions in transport between late endosomes and the trans Golgi network. EMBO J..

[46] Sharma D.K., Choudhury A., Singh R.D., Wheatley C.L., Marks D.L., Pagano R.E. (2003). Glycosphingolipids internalized via caveolar-related endocytosis rapidly merge with the clathrin pathway in early endosomes and form microdomains for recycling. J. Boil. Chem..

[47] Cheryl Chia P.Z., Gleeson P.A. (2011). The Regulation of Endosome-to-Golgi Retrograde Transport by Tethers and Scaffolds. Traffic.

[48] Patterson G.H., Hirschberg K., Polishchuk R.S., Gerlich D., Phair R.D., Lippincott-Schwartz J. (2008). Transport through the Golgi apparatus by rapid partitioning within a two-phase membrane system. Cell.

[49] Bonifacino J.S., Glick B.S. (2004). The mechanisms of vesicle budding and fusion. Cell.

[50] Bonifacino J.S., Traub L.M. (2003). Signals for sorting of transmembrane proteins to endosomes and lysosomes. Annu. Rev. Biochem..

[51] Stansell E., Apkarian R., Haubova S., Diehl W.E., Tytler E.M., Hunter E. (2007). Basic residues in the Mason-Pfizer monkey virus gag matrix domain regulate intracellular trafficking and capsid-membrane interactions. J. Virol..

[52] Dostalkova A., Kaufman F., Krizova I., Kultova A., Strohalmova K., Hadravova R., Ruml T., Rumlova M. (2018). Mutations in the Basic Region of the Mason-Pfizer Monkey Virus Nucleocapsid Protein Affect Reverse Transcription, Genomic RNA Packaging, and the Virus Assembly Site. J. Virol..

[53] Sbalzarini I.F., Koumoutsakos P. (2005). Feature point tracking and trajectory analysis for video imaging in cell biology. J. Struct. Boil..

[54] Chenouard N., Smal I., de Chaumont F., Maska M., Sbalzarini I.F., Gong Y., Cardinale J., Carthel C., Coraluppi S., Winter M. (2014). Objective comparison of particle tracking methods. Nat. Methods.

