# Supplementary material for: Mason-Pfizer Monkey Virus Envelope Glycoprotein Cycling and Its Vesicular Co-Transport with Immature Particles
Source: Viruses. 2018 Oct 20;10(10):575. doi: 10.3390/v10100575 (PMC6212865; doi:10.3390/v10100575)
Supplement: Supplementary file 1 [file viruses-10-00575-s001.zip › viruses-330222-supplementary/viruses-330222-supplementary.pdf]

Supplementary information

Section A

To confirm production of mCherryTM and its incorporation into released virions, we performed western blot assay of lysate of HEK 293T cells transfected with pSARMXmCherryTM WT and of released virions. Figure S1 (the upper panel) shows presence of structural polyprotein Gag (Pr78) in the cell lysate and content of mature capsid protein (p27) in virions collected from the medium and purified by ultracentrifugation. The lower panel confirms intracellular production of mCherry Env precursor (Pr86-mCheryTM) and its incorporation into released virions where it is seen in its mature form as gp22 fused with the mCherry label (gp22-mCherryTM).

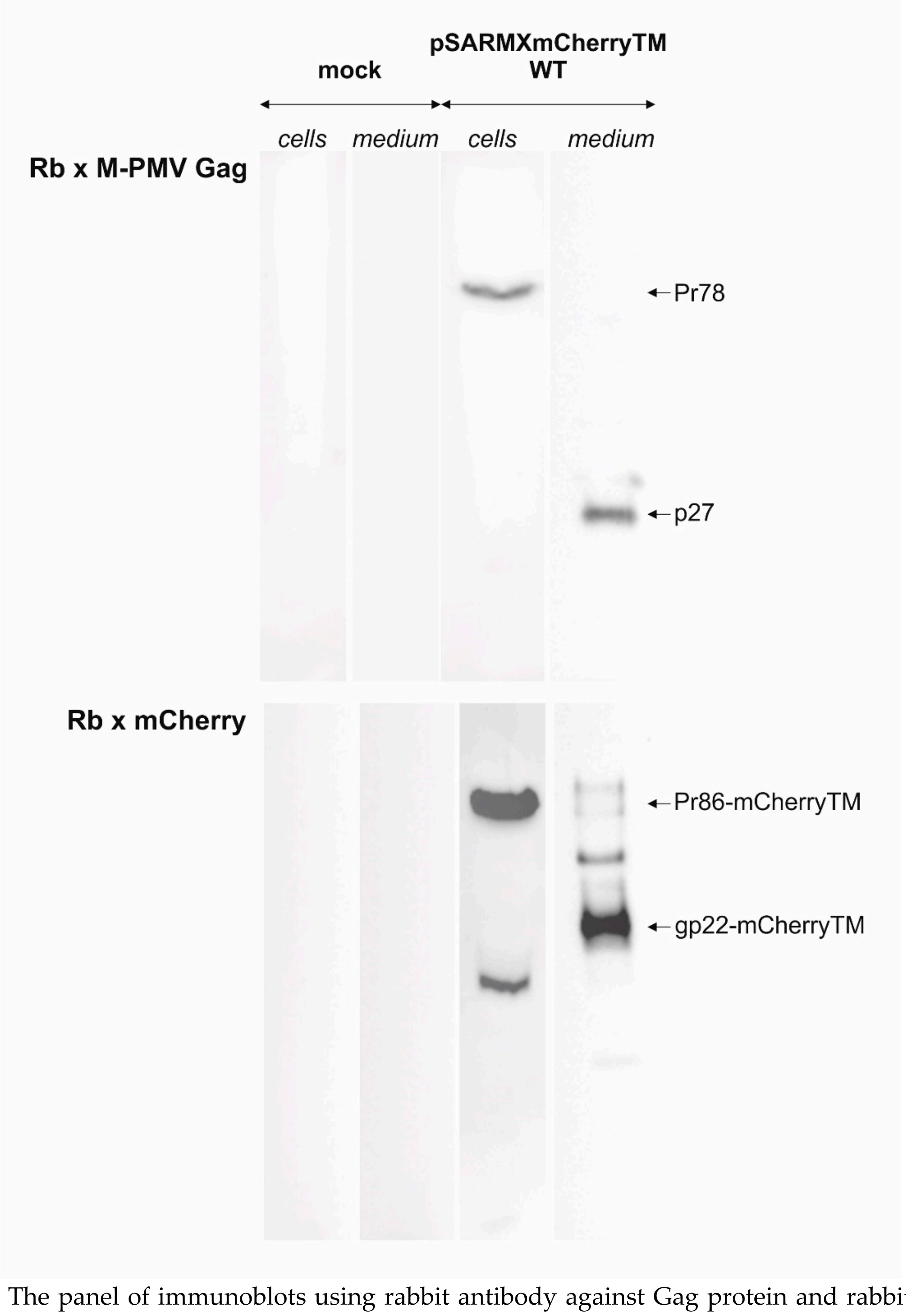

Figure S1: The panel of immunoblots using rabbit antibody against Gag protein and rabbit antibody against mCherry

HEK 293T cells were maintained in Dulbecco's modified Eagle's medium (DMEM, Sigma Aldrich, St. Louis, MO, USA) containing 10% fetal bovine serum. Transfection with pSARMXmCherryTM WT vector (whole M-PMV genome labeled in TM with mCherry) was performed using polyethylenimine (PEI, PolySciences, Warrington, PA, USA) at a 1:2 DNA/PEI ratio. Medium containing released virions was harvested 48 h later. The medium was centrifuged at 250 x RCF, 10 min and filtered through a 0.45 um membrane filter and subsequently ultracentrifuged through a 20% sucrose cushion at 120,000 x RCF, 1 h, 4°C in a Beckman SW28 Ti rotor. The pellets were resuspended in PBS and loaded on the 6-18 % iodixanol gradient (OptiPrep, Sigma Aldrich, St. Louis, MO, USA). The centrifugation was performed at 230,000 x RCF, 1.5 h, 4°C in a Beckman SW41 Ti rotor. The 900 μl fractions of the proper density (determined by refractometry in an interval of 1.11 – 1.17 g/ml; which corresponds to the density of the virions [52]) were combined, diluted and centrifuged at 260,000 x RCF, 1.5 h, 4°C. The pelleted virions were resuspended in protein loading buffer and proteins were separated using SDS-PAGE. Nontransfected cells were processed the same way as a control. The presence of M-PMV proteins as well as mCherry was analyzed by western blot with immunochemical detection using rabbit polyclonal antibody against Gag (in-house production, 1:2,000) and mCherry (1:1,000, Abcam, Cambridge, UK) protein, HRP-conjugated mouse anti-rabbit IgG (1:5,000, Santa Cruz, Dallas, Texas, USA) and chemiluminescent substrate (ECL, BioRad, Hercules, CA, USA).

Section B

Particle tracking measurements were performed using Fiji (version 1.52g, National Institutes of Health, Bethesda, Maryland, USA) plugin Mosaic: Particle tracker 2D/3D (http://mosaic.mpi-cbg.de/?q=downloads/imageJ) [53, 54]. The slope of moment scaling spectrum (MSS) was considered as measure for the type of movement. In case of free and unhindered diffusion, the slope is 0.5. The slope >0.5 indicates directed motion or active transport, while the slope <0.5 implies retardation or immobilization. To determine MSS and its slope, trajectories were generated for randomly selected mCherryTM containing vesicles. For all three mCherry virus variants (WT, I18A and Y22A), two cells from independent experiments were selected. In all analyzed cells, there were at least 6 up to 8 vesicles tracked. The trajectories were filtered to include only those containing at least 50 time points.

Calculated MSS slope values for all evaluated vesicles of all three datasets were plotted into the graphs (Figure S2). According to MSS slope values, all three variants are using active transport. Median of MSS slope for WT is 0.879 (Figure S2, upper panel), for I18A it is 0.8383 (Figure S2, middle panel) and for Y22A it is 0.8325 (Figure S2, lower panel). Additionally, the median of diffusion coefficient was calculated. The value for WT mCherryTM is $8.97x10^{-3}$ $\mu m^2/s$, for I18A mutant it is $1.8x10^{-2}$ $\mu m^2/s$ and for Y22A mutant $3.16x10^{-2}$ $\mu m^2/s$. This parameter shown significant difference by one order of magnitude when compared WT to both mutated variants of mCherryTM.

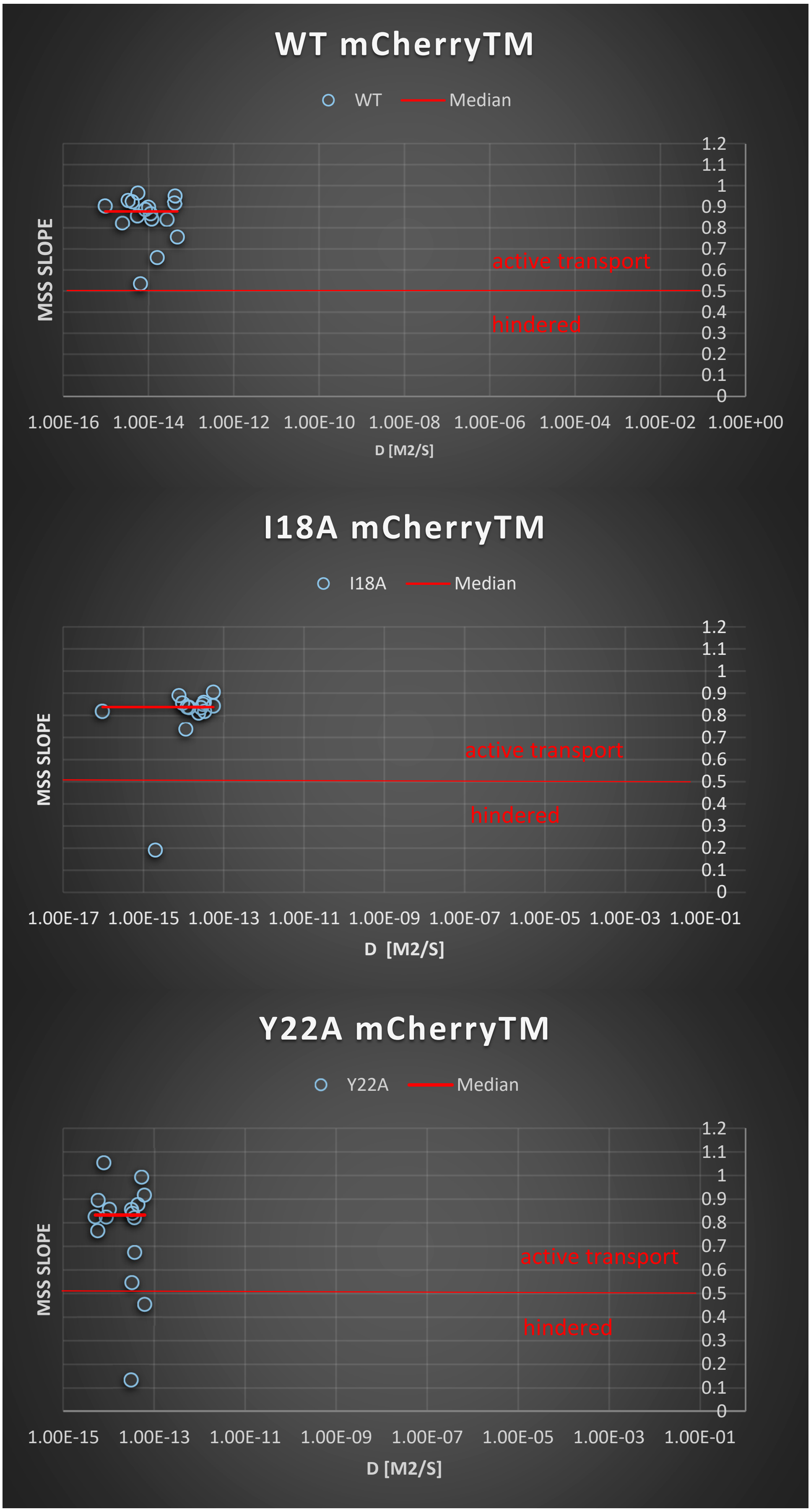

Figure S2 Graphical interpretation of MSS slope values and medians of WT mCherryTM (upper panel), I18A mCherryTM (middle panel) and Y22A mCherryTM (lower panel) containing vesicles,

tracked and analysed via Fiji (version 1.52g, National Institutes of Health, Bethesda, Maryland, USA) plugin Mosaic: Particle tracker 2D/3D. For each variant were two cells from independent experiments chosen and 6 up to 8 vesicles were tracked from each cell. Diffusion coefficient (D) and MSS slope values were plotted in graphs.

References

52. Dostalkova, A., Kaufman, F., Krizova, I., Kultova, A., Strohalmova, K., Hadravova, R., Ruml, T., Rumlova, M. Mutations in the Basic Region of the Mason-Pfizer Monkey Virus Nucleocapsid Protein Affect Reverse Transcription, Genomic RNA Packaging, and the Virus Assembly Site. *Journal of virology* **2018**. *92*.
53. Sbalzarini, I.F., Koumoutsakos, P. Feature point tracking and trajectory analysis for video imaging in cell biology. *Journal of structural biology* **2005**. *151*, 182-195.
54. Chenouard, N., Smal, I., de Chaumont, F., Maska, M., Sbalzarini, I.F., Gong, Y., Cardinale, J., Carthel, C., Coraluppi, S., Winter, M., Cohen, A.R., Godinez, W.J., Rohr, K., Kalaidzidis, Y., Liang, L., Duncan, J., Shen, H., Xu, Y., Magnusson, K.E., Jalden, J., Blau, H.M., Paul-Gilloteaux, P., Roudot, P., Kervrann, C., Waharte, F., Tinevez, J.Y., Shorte, S.L., Willemse, J., Celler, K., van Wezel, G.P., Dan, H.W., Tsai, Y.S., Ortiz de Solorzano, C., Olivo-Marin, J.C., Meijering, E. Objective comparison of particle tracking methods. *Nature methods* **2014**. *11*, 281-289.
